# Semi-Supervised Deep Learning-Based Image Registration Method with Volume Penalty for Real-Time Breast Tumor Bed Localization

**DOI:** 10.3390/s21124085

**Published:** 2021-06-14

**Authors:** Marek Wodzinski, Izabela Ciepiela, Tomasz Kuszewski, Piotr Kedzierawski, Andrzej Skalski

**Affiliations:** 1 Department of Measurement and Electronics, AGH University of Science and Technology, PL30059 Kraków, Poland; skalski@agh.edu.pl; 2 Department of Radiotherapy, The Holycross Cancer Center, PL25734 Kielce, Poland; izabelaciepiela@wp.pl (I.C.); piotr.ke@op.pl (P.K.); 3 Department of Medical Physics, The Holycross Cancer Center, PL25734 Kielce, Poland; tomekq@me.com; 4 Collegium Medicum, Institute of Health Sciences, Jan Kochanowski University, PL25369 Kielce, Poland

**Keywords:** deep learning, image registration, missing data, radiotherapy, breast-conserving surgery

## Abstract

Breast-conserving surgery requires supportive radiotherapy to prevent cancer recurrence. However, the task of localizing the tumor bed to be irradiated is not trivial. The automatic image registration could significantly aid the tumor bed localization and lower the radiation dose delivered to the surrounding healthy tissues. This study proposes a novel image registration method dedicated to breast tumor bed localization addressing the problem of missing data due to tumor resection that may be applied to real-time radiotherapy planning. We propose a deep learning-based nonrigid image registration method based on a modified U-Net architecture. The algorithm works simultaneously on several image resolutions to handle large deformations. Moreover, we propose a dedicated volume penalty that introduces the medical knowledge about tumor resection into the registration process. The proposed method may be useful for improving real-time radiation therapy planning after the tumor resection and, thus, lower the surrounding healthy tissues’ irradiation. The data used in this study consist of 30 computed tomography scans acquired in patients with diagnosed breast cancer, before and after tumor surgery. The method is evaluated using the target registration error between manually annotated landmarks, the ratio of tumor volume, and the subjective visual assessment. We compare the proposed method to several other approaches and show that both the multilevel approach and the volume regularization improve the registration results. The mean target registration error is below 6.5 mm, and the relative volume ratio is close to zero. The registration time below 1 s enables the real-time processing. These results show improvements compared to the classical, iterative methods or other learning-based approaches that do not introduce the knowledge about tumor resection into the registration process. In future research, we plan to propose a method dedicated to automatic localization of missing regions that may be used to automatically segment tumors in the source image and scars in the target image.

## 1. Introduction

### 1.1. Problem Statement

Breast cancer is the most frequently diagnosed cancer among women and the second most common cancer worldwide. More than 2 million new cases were diagnosed in 2018, and more than half a million deaths. Breast cancer accounts for more than a quarter of all malignant tumor cases. Therefore, it is crucial to increase the quality of breast cancer therapy [1,2].

There are several important issues in breast cancer diagnosis and therapy, including early detection, radical surgery and systematic treatment. In the early stages of cancer, breast-conserving surgery (BCS) followed by radiation therapy (RT) is as effective as radical mastectomy. In this work, we focus on the surgical resection proceeded by the supportive RT. The radiation therapy after the BCS decreases the risk of cancer recurrence for all ages [3]. The standard treatment consists of whole breast irradiation with a dose increase for the tumor bed. For a selected group of patients with a lower risk of cancer recurrence, only the tumor bed is irradiated. It is important to precisely estimate the breast tumor bed’s localization (BTB) to minimize the radiation dose delivered to surrounding healthy tissues. There are works discussing advances in precise radiation dose distribution [4]. However, they require precise BTB localization that is a great challenge due to the complex, nonrigid deformations involved.

### 1.2. Related Work

The localization of BTB is a challenging task. First, the breast is a nonrigid structure undergoing large deformations. Second, RT is usually planned using the computed tomography (CT) scans with hardly distinguishable soft tissues. There are significant advances in RT planning using magnetic resonance images (MRI) [4,5]. However, the approach using CT is still widely used in clinical practice. Nevertheless, the CT scans are often acquired before the surgery, which creates a chance to apply image registration (IR) algorithms to find a deformation field mapping the scan acquired before the surgery to the scan acquired after the surgery [6]. The deformation field can then transform the segmented tumor in the preoperative image to localize the BTB in the postoperative scan.

Unfortunately, the BTB localization using IR algorithms is not straightforward, and the traditional algorithms usually fail [6]. The reason is the absence of a breast tumor in the scan acquired after the surgery. This creates the problem of missing data that requires a dedicated approach [7,8,9]. The problem arises because the deformation field calculated by IR algorithms is unable to create new structures. The consequence of this is the non-invertibility of the registration. It is possible to register the scan acquired before the surgery to the scan acquired after the surgery, but not the opposite. The basic methods involve the use of surgical clips that are used as the reference points [10]. However, the surgical clips cannot be placed directly within the tumor bed. Moreover, the surgical clips are rigid structures that are not suited to model complex, nonrigid deformations of the breast [11,12]. The use of symmetric algorithms and enforcing the deformation field to be diffeomorphism can even deteriorate the results. A possible solution to the missing data problem is based on introducing a priori medical knowledge about the resection into the registration process. A summary of the current advances in image registration from both the feature- and the intensity-based perspective can be found in [13,14].

There are several IR algorithms dedicated to the missing data problem. However, they are mainly suited to situation when the missing regions are not the main structures of interest. One of the seminal works introduced locally affine registration resistant to regions without direct correspondences [7]. The method correctly ignores the missing regions but does not address the alignment within them. In [8], the authors proposed an additional dimension to the Demons algorithms [15] that is responsible for automatic localization of the missing structures. Similar to the previous work, the method assumes that the main structure of interest is not missing. Another approach was proposed in [9] where diffusion sources/sinks were introduced to create/delete new structures. This approach is of particular interest. However, the method was not evaluated on any medical data, probably because it is tough to acquire and annotate such a dataset. Moreover, annotating all the missing regions adds another level of complexity.

All the aforementioned works share the same disadvantage, namely the large computational complexity. The classical, nonrigid registration with additional constraints takes a lot of time [16]. In contrast, planning the RT requires the algorithms to be as fast as possible. Ideally, the clinical practice methods should be fully automatic, without any manual annotation of the tumor during the RT planning. These observations bring us to another novel class of IR algorithms based on deep learning (DL).

The DL-based IR is a novel, promising area of research [17]. The main advantage is connected with the fast inference time and transferring all the computational complexity to the training phase. This enables the use of nonrigid IR algorithms in many tasks requiring real-time alignment, e.g., during surgery. There are numerous seminal works about DL-based registration. In [18], the authors introduced a general framework for nonrigid registration, which is currently considered as the state-of-the-art. The authors discussed the deformation fields’ calculation and introduced the segmentation masks to the training phase to guide the registration using a priori medical knowledge. Interestingly, the segmentation masks are not required during the inference, which is a great approach that we share in the work presented in this article. The authors also discussed the possibilities for diffeomorphic registration [19]. A similar approach was introduced in [20] to use the inverse consistency as part of the loss function. Undoubtedly, this approach is correct for many tasks. However, we want to show that enforcing the deformation to be diffeomorphism is not suited for the missing data problems. Another inspiring contribution was presented in [21]. The authors introduced a multilevel algorithm allowing to capture larger deformations which is of particular interest to the registration of the breast. The method significantly improved the registration quality and won one of the IR competitions [22]. There are numerous other influencing contributions like using the generative adversarial networks [23] or proposing frameworks for joint affine and nonrigid registration [24,25]. However, they do not address the problem of missing structures.

### 1.3. Contribution

In this work, we propose a DL-based method dedicated to real-time BTB localization. We hypothesize that addressing large deformations by an appropriate network architecture, together with introducing the a priori medical knowledge into the registration process may improve the registration quality, thus resulting in more precise tumor bed localization. We incorporate the multilevel approach with the proposed, differentiable volume penalty. The volume penalty introduces the knowledge about the tumor resection and performs an implicit tumor segmentation. Therefore, segmentation masks are not required during the inference. We show that the proposed method provides higher registration accuracy than the traditional, iterative methods, as well as DL-based baselines not addressing the missing data problem.

The article is structured as follows. In Section 2, the proposed image registration method, the modified multilevel encoder/decoder architecture, the proposed volume penalty, and the experimental setup, are described. Section 3 presents and qualitative and quantitative results. Section 4 discusses the results and shows the main advantages and limitations or the proposed approach. Finally, the article is summarized in Section 5 and further research directions are highlighted.

## 2. Methods

### 2.1. Overview and Preprocessing

**Figure 1:**
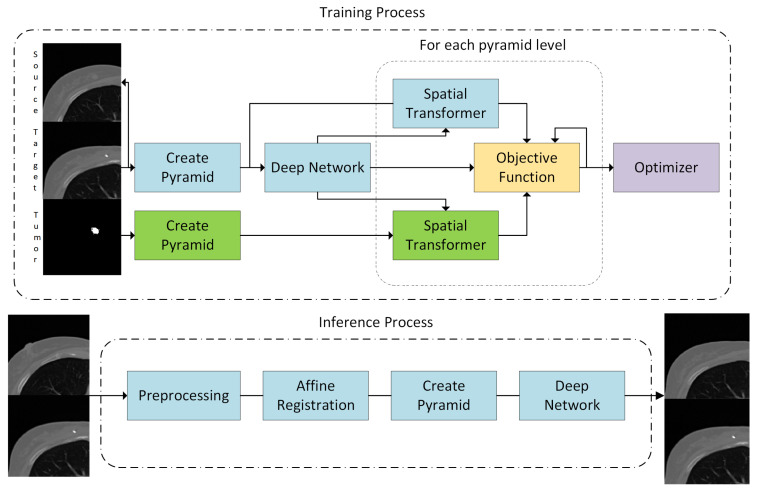
The overview of the proposed method. Note that the segmentation mask is not required during the inference. The volumes are cropped to the region of interest for the presentation clarity. The input to the deep network are images after the affine registration performed before the training process. The training process is described in the text.

**Figure 2:**
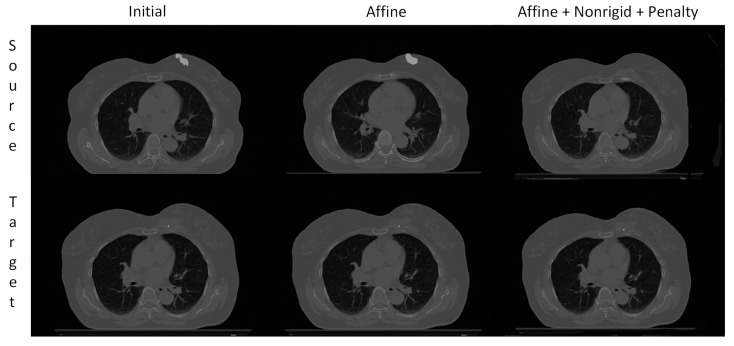
The exemplary visualization after each registration step. The target in the bottom row is repeated for the presentation clarity. The tumor is over-imposed on the source image. Note that the tumor is resected in the target volume and is almost completely missing after the nonrigid registration.

The proposed method consists of several steps: (i) preprocessing, (ii) multilevel, iterative affine registration, (iii) multilevel, DL-based nonrigid registration with the volume penalty. We decided to use the traditional, sequential approach instead of the end-to-end DL-based approach [24] because the affine registration requires a substantially larger amount of training data compared to the following nonrigid step. As a result, large overfitting was observed. Moreover, the iterative affine registration can be performed relatively fast because the input images can be effectively downsampled without decreasing the registration quality. The pipeline is shown in Figure 1, the exemplary visualization of the image after each step is shown in Figure 2.

The preprocessing starts with the intensity values normalization to [0–1] range. Then, the source and target images are resampled to the same isotropic physical spacing equal to 2.0 mm and finally are padded to the same shape. Originally, the images have smaller physical spacing. However, DL methods require a significant amount of memory, and further reduction of the voxel size would exceed the capabilities of the utilized GPU. During training, the preprocessing and the affine registration are done offline to speed-up the process.

### 2.2. Affine Registration

We performed the affine registration using the instance optimization technique [26]. First, a resolution pyramid with 2 levels is created. The isotropic voxel size is equal to 8 mm in the first level and 4 mm in the second level. The process starts with the coarser resolution and initializes the transformation with the identity matrix. Then, transformation is optimized with respect to the cost function using the Adam optimizer with a constant learning rate equal to 0.001. The cost function is the local normalized cross-correlation (NCC) with the window size equal to 7 voxels [18]. The NCC is used because it is the most widely applied similarity measure for unimodal, medical image registration [16]. It is defined as:

(1) $$NCC(M\left(x\right),F\left(x\right))=\frac{1}{N}\sum_{i=1}^{N}\frac{\left(M\left(x_{i}\right)-\mu_{M}\right)\left(F\left(x_{i}\right)-\mu_{F}\right)}{\sigma_{M}\sigma_{F}},$$

where M(x), F(x), $\sigma_{M}$, $\mu_{M}$, $\sigma_{F}$, $\mu_{F}$ denote the moving image, fixed image, standard deviation and mean of intensities in the moving and the fixed image, respectively (within the given window).

The optimization is repeated for each level, starting with the transformation calculated by the preceding level. The process is run until convergence, and the transformation with the largest value of NCC is returned. The affine registration is implemented fully in PyTorch [27] and works on GPU. In studies involving larger training data size, the iterative affine registration can be effectively replaced by the DL-based affine registration to speed-up the process.

### 2.3. Nonrigid Registration Network

**Figure 3:**
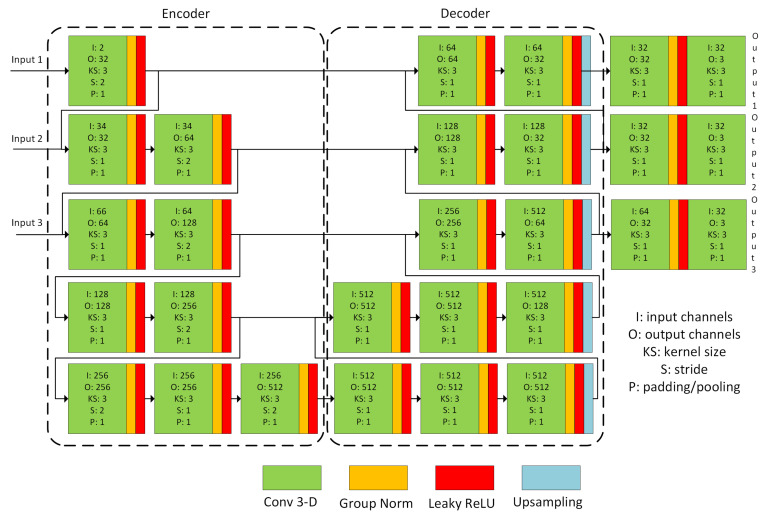
Visualization of the deep network architecture. The arrow connections denote concatenation. The Input 1 denotes the concatenated source/target at the finest resolution level, while the Input 3 is the source/target at the coarsest resolution level. The Output denotes the corresponding displacement field.

The nonrigid registration is performed by a U-Net-like [28] deep neural network with skip connections. The network is presented in Figure 3. The main architectural changes were motivated by the works introduced in [21,29]. The network takes as the input resolution pyramid with 3 levels, concatenates the resampled source/target images, and forwards them to the encoder’s corresponding level. Similarly, the decoder outputs the deformation field at the finest level and for each level of the resolution pyramid. This approach guides the optimizer during training, enabling faster convergence and making it easier to recover larger deformations. Additional modifications of the architecture include the use of group normalization [30] instead of the batch normalization, the use of leaky ReLU, and the trilinear upsampling instead of the transposed convolutions. The group normalization is applied because of the small size of the training batch. The fixed upsampling is used to: (i) diminish the checkerboard artifacts appearing in the calculated deformation field, (ii) an observation that the deconvolution with larger kernel size (divisible by the stride to reduce the artifacts) was harder to train without overfitting.

### 2.4. Unsupervised Training

The nonrigid network is trained mainly in an unsupervised way. The cost function is a weighted sum of the local NCC (window size equal to 5 voxels) and the diffusive regularization term:

(2) J(M,F,u)=S(M∘u,F)+αR(u),

where J(·) is the cost function, S(·) denotes the dissimilarity measure (negative NCC), R(·) denotes the regularization function (diffusive), α is the regularization term controlling the transformation smoothness (700), u denotes the calculated deformation field and M,F are the moving, fixed images respectively.

The training starts with creating a resolution pyramid for a given training case. The resolution pyramid is passed to the multilevel network that outputs displacement fields where each output of the decoder corresponds to a given input level of the encoder. The cost function is evaluated for each pyramid level. The sum of the cost functions forms the loss backwarded during the training. The unsupervised training may be compared to the traditional, iterative formulation of the registration. However, in the learning-based approach, the computation time is transferred to the training phase. Thus, the inference may be performed much faster, enabling real-time nonrigid registration.

The training for each experiment is performed for a predefined number of epochs. The Adam optimizer optimizes the parameters with the initial learning rate equal to 0.002. A scheduler modifies the learning rate with exponential decay equal to 0.97. The images are augmented by small, random affine transformations and intensity value changes.

### 2.5. Volume Penalty

The unsupervised training is supported by the semi-supervised volume penalty, denoting the ratio of the tumor volume after the registration to the tumor volume before the registration. The volume penalty is added to the cost function for each resolution level:

(3) $$J(M,F,u,M_{s})=J(M,F,u)+\frac{V(M_{s}\circ u)}{V(M_{s})},$$

where V(·) is the tumor volume, $M_{s}$ denotes the tumor segmented in the scan acquired before the surgery.

The volume penalty’s motivation is as follows: (i) tumors are fully resected, so the ideal registration should decrease the volume ratio to 0, (ii) the Dice coefficient cannot be applied because the tumor does not exist after the surgery. Notably, the segmentation mask’s warping is performed using the trilinear interpolation because the operation must be differentiable.

### 2.6. Symmetric Registration

We were eager to verify whether our hypothesis that enforcing the deformation field to be a diffeomorphism may decrease the registration quality. To do this, we slightly change the training process and add an additional cost function term [20]. We forward through the network both the source-target pair as well as the target-source pair. Then, the calculated deformation fields are composed, and the inverse consistency is calculated. This term is added to the cost function:

(4) $$\begin{array}{cc} J(M,F,u_{\mathrm{mf}},u_{\mathrm{fm}}) & =J(M,F,u_{\mathrm{mf}})+J(F,M,u_{\mathrm{fm}})\\ \; & +IC(u_{\mathrm{fm}}\circ u_{\mathrm{mf}}), \end{array}$$

where IC(·) is the inverse consistency, $u_{\mathrm{mf}},u_{\mathrm{fm}}$ denote the deformation fields from fixed to moving image and from moving to fixed image respectively.

We verified that this approach improved the registration invertibility. The Jacobian was always positive, which is not true in the tumor region without the inverse consistency term.

### 2.7. Dataset and Experimental Setup

The dataset consists of 30 CT scans acquired before and after the breast tumor surgery. The scans before the surgery were annotated by segmenting the tumor and manually selecting anatomical landmarks close to the breast. The scans after the surgery were annotated only by manual selections of corresponding anatomical landmarks because it is impossible to segment non-existing tumors. In total, there are more than 1000 corresponding landmarks. The acquired scans were anisotropic (average voxel size close to (0.5, 0.5, 2.0) mm), however they were resampled to isotropic voxel size equal to 2 mm due to the GPU memory limitations. The RTX 2080 Ti was the GPU utilized during all the experiments.

The dataset was divided into folds, with the number of folds equal to the number of registration pairs. In each fold, one pair was the test pair not used during the training at all. The remaining pairs were divided into training and validation sets (80% and 20% respectively). The reported results show registration outcome only for the test pairs by combining the results for each fold. This approach was necessary because of the small dataset size and to prove the network generalizability by not using any of the evaluated cases during training. All folds shared the same hyperparameters.

We decided to perform the following experiments: (i) affine registration based on the instance optimization (AR), (ii) AR followed by the classical Demons algorithm (ARD) [31], (iii) AR followed by the nonrigid registration based on the instance optimization (ARRNI) [26], (iv) ARRNI with volume penalty (ARNIP), (v) AR followed by the simple U-Net architecture for nonrigid registration (ARDN) [18], (vi) AR followed by the multilevel U-Net architecture for nonrigid registration (ARDNM) [21], (vii) ARDN with the additional inverse consistency (ARDNI) [20], (viii) ARDN with the volume penalty (ARDNP), (ix) ARDNM with the volume penalty (ARDNMP, proposed in the article). All experiments share the same hyperparameters. Please note that AR, ARD, ARRNI, ARDNM and ARDNI denote the state-of-the-art approaches that are not proposed in this study.

The Demons algorithm is used as the reference because previous studies have shown the most promising result among the classical registration algorithms [6]. The nonrigid registration based on the instance optimization is implemented using PyTorch, similarly to the affine registration. It is run using GPU in the multilevel pipeline. The simple U-Net architecture does not include the multilevel approach and takes as input the source/target pair at the finest resolution level. The source code of the DL experiments is available at [32]. All the experiments and networks are implemented using PyTorch library [27].

## 3. Results

The proposed contributions are compared to the state-of-the-art using both the quantitative (target registration error (TRE), tumor volume ratio (TVR)) and the qualitative visual assessment. The results are presented in the following subsections and discussed in Section 4.

### 3.1. Target Registration Error

The TRE is one of the evaluation metrics. It is defined as:

(5) $$TRE\left(M_{l},F_{l}\right)=\sqrt{\sum_{i=1}^{3}{\left(M_{l}\left(i\right)-F_{l}\left(i\right)\right)}^{2}}$$

where $M_{l},F_{l}$ are the warped moving landmarks and fixed landmarks respectively.

**Figure 4:**
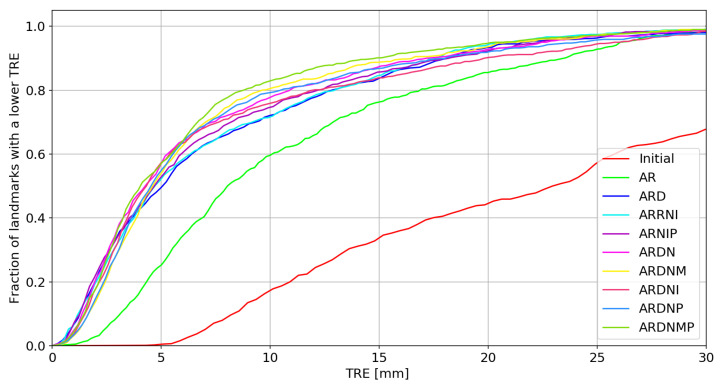
The cumulative histogram of the TRE for landmarks close to the tumor. Note that both the volume penalty and the multilevel approaches decreases the TRE. Abbreviations are described in the text.

**Figure 5:**
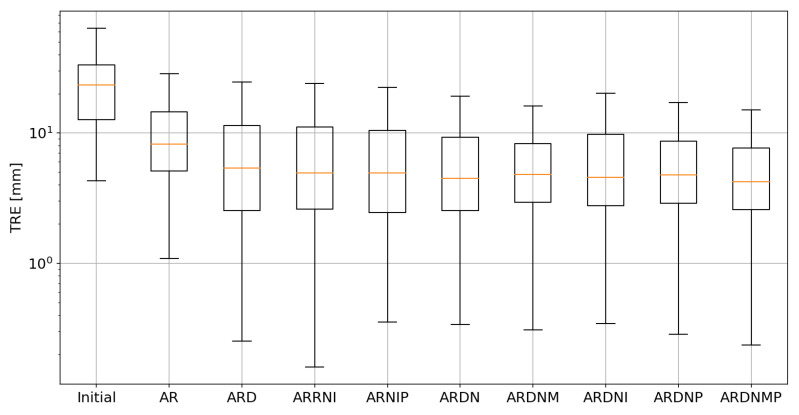
The box plot presenting the TRE for landmarks close to the tumor. The influence of the volume penalty on the TRE is limited due to the small size of the tumor. However, the reduction can be observed both for the multilevel approach, as well as introducing the penalty. Abbreviations are described in the text.

The TRE, together with the Dice coefficient, are the most frequently applied evaluation metrics in the medical image registration [33]. However, the situation in the presented study is quite different. The TRE should be interpreted with care, keeping in mind that the correspondence cannot be directly estimated between the tumor in the moving image and its bed in the fixed image. The cumulative histogram presenting the TRE for different methods is shown in Figure 4. The box plot showing the TRE is presented in Figure 5. The TRE is also summarized in Table 1.

### 3.2. Tumor Volume Ratio

**Figure 6:**
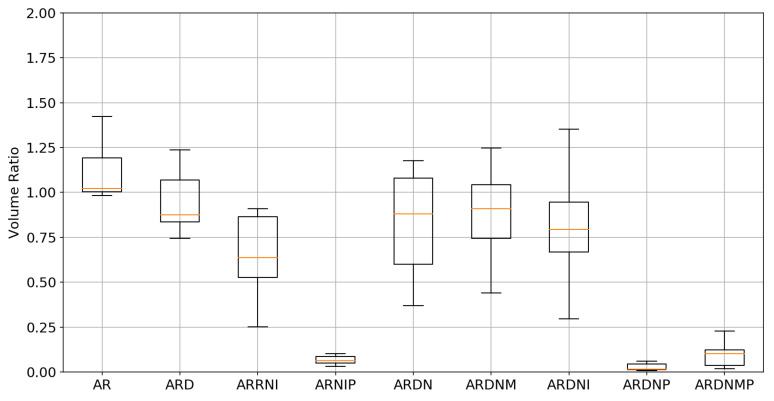
The box plot presenting the tumor volume ratio. Note that the volume penalty significantly decreases the tumor volume. The value for the multilevel approach is slightly increased due to the non-ideal interpolation at lower pyramid levels. Interestingly, without the penalty term, for few cases, the tumor volumes increase, which is inherently wrong.

Another evaluation metric is the TVR, defined as in Equation (3). The tumor is fully resected during the BCS. Therefore, the tumor volume ratio should be equal to zero. This fact can be used during the registration to guide the process and evaluate the registration correctness. It is crucial to emphasize that the TVR equal to zero does not imply a perfect registration, but TVR greater than zero for sure implies imperfect registration. Therefore, the TVR should be combined with the TRE because decreasing the TVR should also be reflected in a small TRE decrease. The TVR for different experiments is shown in Figure 6 and in Table 1.

### 3.3. Visual Assessment

**Figure 7:**
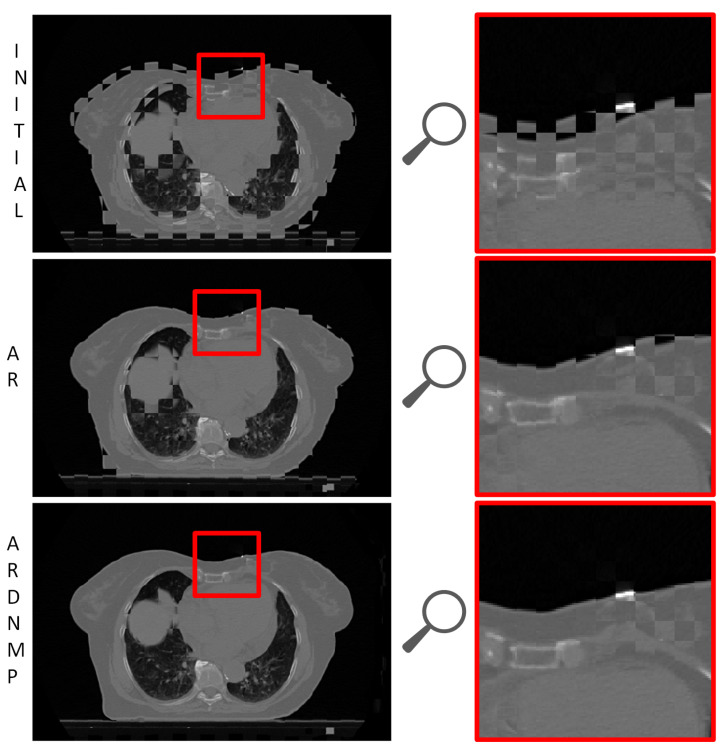
The checkerboard presenting exemplary registration results. In the right column the visualization is zoomed to the region of interest containing the tumor in the source image and its bed in the target image.

The visual assessment is crucial to evaluate the medical image registration algorithms. It allows easy verification of medically implausible deformations. In Figure 2, we show the volumes after subsequent registration stages, and in Figure 7, we present the checkerboards. The checkerboards are zoomed to the region of interest containing the tumor in the source image.

## 4. Discussion

The presented results show that both the multilevel approach and the volume penalty improve the registration results. Both the TRE and TVR decrease after the registration. As mentioned in the results section, the TRE or TVR alone cannot evaluate the image registration with missing data. However, the joint decrease of TRE and TVR shows that the corresponding structures near the tumor bed get closer, which indicates the registration quality. The changes in TRE thanks to the volume regularization are not that significant because breast tumors resected by lumpectomy or tumorectomy are usually diagnosed in the early stage, and their physical size is not that high. The visual assessment of the registration methods confirms the quantitative results. The results are credible, and the alignment is improved.

An interesting observation can be made for the registration based on the instance optimization. The TRE indicates that the registration is more accurate in recovering small deformations. This agrees with the intuition because during the instance optimization the loss function is optimized separately for each case. Simultaneously, in the DL-based approach, the knowledge is generalized across all previously seen cases. Therefore, the instance optimization has a better ability to recover subtle details compared to the learning-based solution. Noteworthy, the instance optimization requires the tumor to be segmented before the registration. In contrast, the learning-based approach learns how to segment the tumor implicitly, and the segmentation mask is not necessary during the inference.

The registration time is significantly lower for the DL-based approach. The registration is more than an order of magnitude faster than the instance optimization implemented on GPU and more than two orders of magnitude faster than the CPU implementation of the Demons algorithm. It shows the great potential of learning-based solutions for real-time radiotherapy planning.

One could argue that the TVR should not be used as the evaluation criteria since it is used as the penalty term during training. This is not true since the same argument applies to using the Dice score as the loss function for the segmentation tasks. The tumor mask is not used during the inference. Therefore the TVR correctly evaluates the network’s ability to generalize into unseen data and to localize the missing region implicitly. In future work, we plan to verify whether this approach can implement label-less segmentation of all missing regions by combining the volume penalty with automatically generated heatmaps, similarly to the attention-based networks used for classification tasks [34].

Nevertheless, the study has several limitations. As mentioned in the Methods section, the tumor is warped using a trilinear interpolation to make the loss differentiable. This is not a perfect approach since this kind of interpolation may introduce changes to the tumor topology, resulting in an incorrect registration [35]. All tumors in this study have regular shapes and do not suffer from this issue. However, this is an important factor to consider in further research. It would be interesting to propose a topology-preserving warping that is differentiable and useful for the learning-based algorithms.

Another limitation of the study is connected with the training time of the deep network. Performing the presented experiments has taken more than 3 months of continuous training using RTX 2080 Ti. The training time was significantly increased by utilizing many folds and performing ablation studies verifying the influence of a particular step on the registration results. Nevertheless, it is arguable whether it makes sense to train a network, e.g., 2 days to address just several tumor cases. A single model that can localize the tumor bed independently of the equipment used for the acquisition would be a great tool for radiologists, enabling real-time radiotherapy planning.

This brings us to further possibilities. It would be interesting to gather data from several institutions and evaluate the algorithm on a larger, heterogeneous dataset. Perhaps a domain adaptation using generative adversarial networks could enable standardization of the acquired scans and make the method useful, independently of the scanner used for the acquisition [36].

The proposed method uses a relatively large voxel size equal to 2 mm × 2 mm × 2 mm. The reason for this is the limited memory available in RTX 2080 Ti (11 GB). The GPU architectures’ constant progress (e.g., RTX 3090 has 24 GB of available memory) will enable significantly smaller voxel size and hopefully more accurate registration.

Noteworthy, the method may be useful for other tumors. Perhaps it will require a different model for each tumor type. However, the same approach may be utilized for any structure that after removal requires supportive radiotherapy.

In future work, we plan to perform a medical study on a larger scale, comparing the results acquired with the presented method with the radiologists’ dose distributions. Moreover, we want to address scar creation. Not only is the tumor missing, but also the scar is introduced that is absent in the scan acquired before the surgery.

## 5. Conclusions

To conclude, we propose a novel, semi-supervised DL-based image registration method dedicated to tumor bed localization. The proposed method improves the registration accuracy in terms of the target registration error, tumor volume ratio, and subjective visual assessment. We freely release the source code containing the DL-based experiments to improve the reproducibility of the results. The method may be useful for real-time, supportive radiotherapy planning to decrease the radiation dose delivered to healthy tissues surrounding the resected tumor and, as a result, decreasing the risk of secondary carcinogenesis. In future research, we plan to propose a method dedicated to automatic localization of regions with missing correspondences, and to perform a large scale medical study that compares the dose distributions defined using the presented method with the dose distributions proposed by radiologists.

## Figures and Tables

**Table 1:** Table summarizing the TRE, TVR, and the registration time. The average time reports the time required for the data loading, preprocessing, initial alignment, and the nonrigid registration. Note that the nonrigid network’s inference time is an order of magnitude faster than the instance optimization or the Demons algorithm. Abbreviations are described in the text.

Experiment	Average TRE [mm]	Median TRE [mm]	Average TVR	Median TVR	Average Time [s]
*Initial*	*24.47*	*23.32*	*1.00*	*1.00*	*-*
AR	*10.88*	*8.22*	*1.10*	*1.02*	*0.34*
ARD	*7.86*	*5.35*	*0.96*	*0.88*	*51.18*
ARRNI	*7.60*	*4.95*	*0.69*	*0.63*	*4.15*
ARNIP	*7.50*	*4.92*	*0.07*	*0.06*	*4.78*
ARDN	*7.45*	*4.75*	*0.82*	*0.88*	*0.52*
ARDNM	*7.07*	*4.80*	*0.88*	*0.90*	*0.54*
ARDNI	*7.78*	*4.56*	*0.81*	*0.79*	***0.51***
ARDNP	*7.15*	*4.49*	***0.03***	***0.01***	*0.53*
**ARDNMP**	***6.51***	***4.22***	*0.10*	*0.10*	*0.54*

## Data Availability

The dataset is available from I.C., P.K., T.K. on reasonable request and after signing an appropriate agreement.

## Abbreviations

The following abbreviations are used in this manuscript:

BCS	Breast-Conserving Surgery
BTB	Breast Tumor Bed
CPU	Central Processing Unit
CT	Computed Tomography
DL	Deep Learning
GPU	Graphics Processing Unit
IR	Image Registration
MRI	Magnetic Resonance Images
NCC	Normalized Cross-Correlation
RT	Radiation Therapy
TRE	Target Registration Error
TVR	Tumor Volume Ratio
